# Associations With Baseline Blood Pressure Control in NURTuRE-CKD

**DOI:** 10.1016/j.ekir.2024.02.013

**Published:** 2024-02-15

**Authors:** Bethany J. Lucas, Paul Cockwell, Simon D.S. Fraser, Philip A. Kalra, David C. Wheeler, Maarten W. Taal

**Affiliations:** 1 Centre for Kidney Research and Innovation, Academic Unit for Translational Medical Sciences, School of Medicine, University of Nottingham, Nottingham, UK; 2 Department of Renal Medicine, Royal Derby Hospital, University Hospitals of Derby and Burton NHS Foundation Trust, Derby, UK; 3 Department of Renal Medicine, Queen Elizabeth Hospital, Birmingham, Institute of Ageing and Immunity, University of Birmingham, Birmingham, UK; 4 School of Primary Care, Population Sciences and Medical Education, Faculty of Medicine, University of Southampton, Southampton, UK; 5 Salford Royal Hospital, Northern Care Alliance NHS Foundation Trust, Salford, UK; 6 Department of Renal Medicine, University College London, London, UK

**Keywords:** albuminuria, antihypertensives, chronic kidney disease, hypertension

## Introduction

Hypertension is commonly associated with chronic kidney disease (CKD) and is a key mediator of progressive kidney damage and the associated increase in cardiovascular mortality.^1^ The importance of blood pressure (BP) control as a therapeutic intervention in CKD, both for reduction in mortality and progression of kidney disease, is well-established,^2^^,^^3^ but many patients do not achieve their BP targets.^4^ The optimum BP target is debated, especially following the cardiovascular benefit shown by targeting a lower systolic BP of <120 mm Hg in the Systolic BP Intervention Trial (SPRINT).S1

The National Unified Renal Translational Renal Enterprise (NURTuRE) CKD is a prospective cohort study of participants from secondary care nephrology centers in the UK, which aims to study risk factors for adverse outcomes associated with CKD.^5^ In this analysis, we assessed BP control at baseline against targets recommended by 3 different guidelinesS2–S4 and investigated factors associated with BP control in order to identify subgroups who may benefit from additional clinical input.

## Results

### Study Population

Of the 2996 participants, 2683 with available baseline estimated glomerular filtration rate, urinary albumin-to-creatinine ratio, BP readings, and medication history were included in this analysis (Supplementary Methods). Of these participants, 59.3% were male, 86.6% were of White ethnicity, and 30.3% had diabetes. Mean±SD estimated glomerular filtration rate was 37±18 ml/min per 1.73 m^2^ and median (interquartile range, IQR) urinary albumin-to-creatinine ratio was 211 (33–938) mg/g. Median age (IQR) was 65 (53–73) years. For those with available data (2624/2683), median (IQR) time registered in secondary care nephrology was 4 (3–6) years.

### BP Control

Mean baseline systolic BP for the cohort was 140±20 mm Hg and diastolic BP was 80±12 mm Hg. Analysis of BP in clinically important subgroups is shown in Supplementary Table S1. A higher mean systolic BP was observed with age over 65 years compared to those under 65 years, whereas mean diastolic BP was lower in those over 65 years. Mean systolic BP was also higher in males than in females, and in Black ethnicity than in White, Asian, and other ethnicities. Lower estimated glomerular filtration rate categories, participants with diabetes, and those in higher albuminuria categories or higher body mass index category also had higher mean systolic BP. For those prescribed renin-angiotensin system inhibitors, diastolic BP was significantly higher. Current smokers had a significantly higher diastolic BP compared with ex-smokers and nonsmokers.

In Supplementary Figure S1, we show the median (IQR) BP by Kidney Disease Improving Global Outcomes (KDIGO) heat map category. For those in the high risk (red) KDIGO categories mean systolic BP was 142 ± 21 mm Hg versus 134±18 mm Hg in lower risk categories (green, yellow, and amber) (*P* < 0.001). For the lowest risk category (green), mean systolic BP was 133 ± 18 mm Hg; for low risk (yellow), it was 134±18 mm Hg, and for medium risk (orange), it was 135±19 mm Hg (Supplementary Figure S2).

BP control by guideline target is shown in Table 1. For the 2014 National Institute for Health and Care Excellence (NICE) guideline 37.8% of participants achieved target BP. For KIDGO 2012 and 2021 guidelines, 30.3% and 15.2% achieved BP control, respectively. The proportion of participants achieving their target was lower in higher albuminuria categories. Target achievement by KDIGO category is shown in Supplementary Figures S3–S5.

**Table 1:** Mean blood pressure and proportion of participants in different categories achieving BP control according to different guideline targets in NURTuRE-CKD

	Diabetes, *n* = 812			No Diabetes *n* = 1871			Total *N* (%)
**Albuminuria status**	**A1*****n*** **= 140**	**A2*****n*** **= 248**	**A3*****n*** **= 424**	**A1*****n*** **= 485**	**A2*****n*** **= 629**	**A3*****n*** **= 757**	***n*** **= 2683**
Mean systolic BP (mm Hg)	134 ± 18	140 ± 21	149 ± 21	134 ± 19	137 ± 20	141 ± 20	140 ± 20
Mean diastolic BP (mm Hg)	72 ± 11	75 ± 11	79 ± 13	79 ± 11	81 ± 12	84±12	80 ± 12
**Albuminuria status**	**A1*****n*** **= 140**	**A2*****n*** **= 248**	**A3*****n*** **= 424**	**ACR <70mg/mmol*****n*** **= 1323**		**ACR ≥70mg/mmol*****n*** **= 548**	***n*** **= 2683**
BP controlled (NICE 2014 target)^a^	55 (39.3)	63 (25.4)	58 (13.7)	748 (56.5)		89 (16.2)	1013 (37.8)
BP controlled (KDIGO 2021 target)^b^	31 (22.1)	38 (15.2)	26 (6.1)	107 (22.0)	116 (18.4)	91 (12.0)	409 (15.2)
BP controlled (KDIGO 2012)^c^	89 (63)	63 (25.4)	58 (13.7)	300 (61.9)	164 (26.1)	138 (18.2)	812 (30.3)

ACR, albumin-to-creatinine ratio; BP, blood pressure; CKD, chronic kidney disease; KDIGO, Kidney Disease Improving Global Outcomes; NICE, National Institute for Health and Care Excellence.

Data are presented as n (%) or mean ± SD.

a NICE 2014 <140/90 mm Hg without diabetes, <130/80 mm Hg with diabetes or ACR ≥70mg/mmol.

b KDIGO 2021 - <120 mm Hg systolic.

c KDIGO 2012 <140/90 mm Hg, unless high risk ACR >30 mg/g then <130/80 mm Hg.

### Antihypertensives

The median number of antihypertensive agents prescribed was 2 (IQR, 1–3), with 2408 (89.8%) participants prescribed at least 1 antihypertensive or diuretic agent; 679 (25.3%) participants were prescribed a single agent, 699 (26.1%) were prescribed 2 agents, and 1030 (38.4%) were prescribed 3 or more agents. Of those prescribed antihypertensives, 1830 (68.2%) were prescribed either an angiotensin receptor blocker or angiotensin-converting enzyme inhibitor. In the highest albuminuria category 914 (77.4%) were prescribed an angiotensin-converting enzyme inhibitor or angiotensin receptor blocker. For participants in the high risk (red) KDIGO categories (*n* = 2058), 847 of participants (41.2%) were prescribed 3 or more agents, whereas 159 (18.8%) were prescribed none. The second most common class of antihypertensive was calcium channel blockers (*n* = 1233, 46%) followed by beta-blockers (*n* = 882, 32.9%). Thiazide diuretics were prescribed in 334 (12.4%) of those prescribed antihypertensives and alpha blockers in 23.0%. The distribution of antihypertensive combinations is illustrated in Supplementary Figure S6.

Those aged 65 years and over were prescribed on average 2.3±1.4 antihypertensives compared with 2.0 ± 1.4 in the younger age group (*P* < 0.001 for difference). In the older age group, the mean BP for those prescribed 3 or more antihypertensives was 146 ± 21 mm Hg systolic and 75±12 mm Hg diastolic.

Mean systolic BP was higher in those prescribed a greater number of antihypertensives (Supplementary Figure S7.): 144±21 for those prescribed 3 or more antihypertensives versus 133±19 for those prescribed none (*P* = <0.001). Of those prescribed at least 3 agents, 109 participants (10.5%) achieved control by KDIGO 2021 target, 236 (23%) by KDIGO 2012, and 288 (28%) for the NICE guidelines.

### Factors Associated With BP Control

In univariable analysis (Supplementary Table S2.) diabetes, body mass index >30 m/kg^2^, taking 3 or more antihypertensives, lower estimated glomerular filtration rate, and higher albuminuria category were all associated with a lower odds ratio of achieving BP target across all 3 guidelines. In the KDIG0 2012 and NICE guidelines, male sex and a history of atherosclerotic cardiovascular disease were also associated with a lower odds ratio of achieving the target. For KDIGO 2021 and NICE, aged ≥65 years was associated with lower odds ratio of BP control.

In multivariable analysis, being aged 65 years or older, having a body mass index >30 m/kg^2^, prescribed 3 or more antihypertensives and albuminuria category A3 were associated with lower odds ratio of achieving target across all 3 guidelines (Table 2.). A2 category albuminuria was significantly associated with lower odds ratio of control for KIDGO 2012 and NICE 2014. In contrast, there were no significant associations with sex, ethnicity, or educational status. A history of diabetes was only significantly associated with lower odds of BP control for the NICE 2014 where <130/80 mm Hg was the target for those with diabetes. A history of cardiovascular disease and being in the lowest index of multiple deprivation was associated with increased odds of achieving the lower KDIGO 2021 target.

**Table 2:** Multivariable associations with BP control by guideline

Characteristics		Multivariable odds ratio of achieving KDIGO 2012	*P*	Multivariable Odds ratio of achieving KDIGO 2021	*P*	Multivariable Odds ratio of achieving NICE	*P*
		OR (95% CI)		OR (95% CI)		OR (95% CI)	
Age, yr	≥65	0.61 (0.44, 0.84)	0.002	0.46 (0.31, 0.68)	<0.001	0.60 (0.44, 0.81)	<0.001
	<65	Reference		Reference		Reference	
Sex	Male	0.95 (0.78, 1.16	0.625	1.07 (0.84 ,1.35)	0.602	0.96 (0.79 ,1.15)	0.633
	Female	Reference		Reference		Reference	
Ethnicity	Non-White ethnicity	1.02 (0.76, 1.38)	0.875	1.13 (0.79, 1.60)	0.506	1.07 (0.81, 1.42)	0.630
	White ethnicity	Reference		Reference		Reference	
Diabetes	Diabetes	1.18 (0.94, 1.48)	0.163	0.98 (0.74, 1.30)	0.886	0.482 (0.39, 0.60)	< 0.001
	No diabetes	Reference		Reference		Reference	
BMI (m/kg^2^)	>30	0.63 (0.48, 0.81)	<0.001	0.52 (0.39, 0.70)	<0.001	0.67 (0.52, 0.85)	<0.001
	25–30	0.65 (0.51, 0.84)	<0.001	0.62 (0.46 ,0.82)	<0.001	0.72 (0.57, 0.91)	0.006
	<25	Reference		Reference		Reference	
Smoking Status	Ever smoked	1.06 (0.87, 1.29)	0.543	1.11 (0.88, 1.40)	0.395	1.12 (0.94, 1.35)	0.210
	Never smoked	Reference		Reference		Reference	
History of CVD disease	Yes	1.22 (0.931, 1.59)	0.151	1.43 (1.03, 1.98)	0.031	1.214 (0.94, 1.57))	0.137
	No	Reference		Reference		Reference	
Employment	Working	Reference		Reference		Reference	
	Retired	1.16 (0.85, 1.59)	0.355	1.14 (0.78, 1.67)	0.504	1.00 (0.74, 1.35)	0.998
	Unemployed	1.38 (0.73, 2.59)	0.318	1.40 (0.71, 2.75)	0.336	0.95 (0.51, 1.76)	0.860
	Student	2.57 (0.61, 10.91)	0.200	1.81 (0.41, 7.91)	0.430	4.22 (0.80,22.13)	0.089
	Other	0.62 (0.43, 0.90)	0.013	0.78 (0.52, 1.18)	0.242	0.714 (0.510, 1.000)	0.037
Education status	No qualifications	Reference		Reference		Reference	
	GCSE	1.08 (0.82, 1.43)	0.590	0.94 (0.67, 1.32)	0.703	1.11 (0.85, 1.44)	0.450
	A Levels	0.96 (0.64, 1.46)	0.860	1.035 (0.64, 1.68)	0.890	1.07 (0.73, 1.56)	0.741
	NVQ	0.98 (0.6, 1.37)	0.905	1.01 (0.74, 1.62)	0.638	0.96 (0.71, 1.32)	0.815
	First degree	1.23 (0.89, 1.68)	0.206	0.98 (0.66, 1.43)	0.900	1.13 (0.84, 1.52	0.408
	Higher degree	1.38 (0.97, 1.96)	0.078	0.95 (0.61, 1.49)	0.836	1.41 (1.01, 1.97)	0.044
	Other	0.94 (0.26, 3.42)	0.921	0.46 (0.06, 3.67)	0.462	1.09 (0.33, 3.57)	0.885
IMD Quintiles	1 (Most deprived)	0.84 (0.62, 1.15)	0.283	0.56 (0.38, 0.82)	0.003	0.94 (0.70, 1.26)	0.681
	2	0.98 (0.72, 1.34)	0.920	0.74 (0.51, 1.06)	0.103	0.94 (0.70, 1.25)	0.668
	3	0.92 (0.68, 1.26)	0.612	1.024 (0.72, 1.46)	0.894	0.94 (0.70, 1.27)	0.697
	4	1.06 (0.78, 1.4)	0.715	0.9 (0.63, 1.28)	0.560	1.08 (0.81, 1.44)	0.606
	5 (least deprived)	Reference		Reference		Reference	
Number of antihypertensives	None	Reference		Reference		Reference	
	1	0.90 (0.62, 1.32)	0.600	0.93 (0.60, 1.43)	0.742	0.93 (0.65, 1.33)	0.684
	2	0.90 (0.61, 1.34)	0.610	0.75 (0.47, 1.18)	0.215	0.95 (0.66,1.38)	0.791
	3 or more	0.61 (0.41, 0.93	0.020	0.55 (0.34, 0.90)	0.017	0.66 (0.45, 0.97)	0.032
RAASi	No	Reference		Reference		Reference	
	Yes	1.15 (0.90,1.48)	0.256	1.21 (0.89,1.64)	0.225	1.22 (0.97,1.54)	0.094
UACR mg/g	A1	Reference		Reference		Reference	
	A2	0.20 (0.16, 0.25)	<0.001	0.76 (0.58, 1.01)	0.054	0.67 (0.54, 0.84)	< 0.001
	A3	0.11 (0.09, 1.4)	<0.001	0.34 (0.25, 0.46)	< 0.001	0.19 (0.15, 0.24)	< 0.001
eGFR, ml/min per 1.73 m^2^	Per 1 ml/min per 1.73 m^2^	1.00 (0.99, 1.00)	0.225	1.00 (0.99, 1.01)	0.991	1.00 (0.99, 1.00)	0.120

BMI, body mass index; CI, confidence interval; CVD, cardiovascular disease; eGFR, estimated glomerular filtration rate; GCSE, general certificate of secondary education; IMD, index of multiple deprivation; KDIGO, Kidney Disease Improving Global Outcomes; NICE, National Institute for Health and Care Excellence; NVQ, national vocational qualification; RAASi, renin-angiotensin system inhibition; UACR, urinary albumin-to-creatinine ratio.

## Discussion

This analysis demonstrates suboptimal BP control among people with CKD when compared to all 3 major guidelines with only a minority (15.2%–37.8%) of participants within target. In multivariable analyses, age ≥65 years, body mass index >30 kg/m^2^, taking 3 or more antihypertensives and higher albuminuria category were associated with poorer BP control across all guidelines. Proteinuria and higher age have previously been associated with poorer control, as has male sex which was not significant in our analysis.S5–S6

We also observed that those in the highest risk categories demonstrated poorer control. The reasons for this are not clear but may include treatment resistant hypertension in CKD, medication nonadherence, or fear of polypharmacyS12. Given the higher risk of CKD progression and cardiovascular mortality in this group,^1^^,^^6^^,^^7^ addressing the disparity between target and achieved BP is paramount.

Strengths of this analysis include a large study population from across the UK with varying causes of CKD and a large proportion in high risk KDIGO categories. BP measurements were taken according to a standardized operating procedure, similar to the technique recommended in international guidelines and consensus documents.^8^^,^^9^ Nevertheless, our findings should be considered in the context of some limitations. Importantly, BP was only measured at a single baseline visit. More robust observation would likely be obtained with repeated measurements, home BP measurements, or 24-hour ambulatory measurements. Medication data was collected from electronic health records and self-reported by participants at baseline visits and no measure of medication concordance was recorded. However, the study design reflects the “real world” situation and has identified high risk subgroups that will inform focused interventions for improving BP control. Finally, the study was performed in secondary care with volunteers in the UK and may not be representative of other populations.

Given the importance of BP control as the fundamental intervention to improve outcomes in CKD, further research is warranted to understand the reasons for poor BP control and to develop strategies for improvement with initial focus on those aged ≥65 years, with obesity, or with more severe albuminuria.

## Disclosure

MWT reports consulting fees from Boehringer Ingelheim, honoraria from Bayer and support to attend conferences from Bayer, and a leadership role in the International Society of Nephrology. BJL reports grant funding from the National Institute for Health Research. PC reports a leadership position in the UK Kidney Association. DCW reports grant funding from Kidney Research UK; consultancy fees from Astellas, AstraZenca, Bayer, Boehringer Ingelheim, GlaxoSmithKline, Gilead, Janssen, ProKidney and Tricida; honoraria from Amgen, Mundipharma, Merck Sharp and Dohme, and Zydus; support for attending meetings from Astellas and AstraZeneca; participation in the data safety monitoring board for the following studies: ProKidney, Galderma, and Eledon; and a leadership role in the International Society of Nephrology; SDSF reports grant funding from Kidney Research UK. PAK reports grant funding from Vifor and Astellas; consulting fees from Astra Zeneca, Vifor, Unicyte, and UCB; honoraria from Vifor, Astra Zeneca, and Pfizer; and support for attending meetings from Pharmacosmos and Vifor. All the other authors declared no competing interests.

## Supplementary Material

**Supplementary Methods.**

**Figure S1.** Blood pressure in the NURTuRE-CKD cohort according to KDIGO CKD risk categories (*n* = 2683, data are median [IQR]).

**Figure S2.** Mean systolic and diastolic BP by number of antihypertensives.

**Figure S3.** Venn diagram showing the pattern of use of the three most commonly prescribed anti-hypertensives at baseline in the NURTuRE-CKD cohort.

**Figure S4.** Mean ± standard deviation BP at baseline by KDIGO CKD risk categories in the NURTuRE CKD cohort study (*n* = 2683).

**Figure S5.** Proportion of participants meeting KDIG0 2021 BP guideline (systolic BP <120 mmHg) by KDIGO GFR category *n* (%) in NURTuRE CKD at baseline >120 mmHg) by KDIGO GFR category *n* (%) in NURTuRE CKD at baseline.

**Figure S6.** Proportion of participants meeting KDIGO 2012 BP target (<140/90 mmHg, unless high risk ACR >30 mg/g then <130/80 mmHg) in NURTuRE CKD at baseline.

**Figure S7.** Proportion of participants meeting NICE BP target (<140/90 mmHg without diabetes, <130/80 mmHg with diabetes or uACR ≥ 70 mg/mmol) in NURTuRE CKD.

**Table S1.** Blood Pressure by baseline demographics.

**Table S2.** Univariate associations with BP control by guideline.

BP, blood pressure

**Supplementary Reference**.

**STROBE statement**.

## References

[1] Matsushita K., van der Velde M. (2010). Association of estimated glomerular filtration rate and albuminuria with all-cause and cardiovascular mortality in general population cohorts: a collaborative meta-analysis. Lancet.

[2] Ninomiya T., Perkovic V., Perkovic V. (2013). Blood pressure lowering and major cardiovascular events in people with and without chronic kidney disease: meta-analysis of randomised controlled trials. BMJ.

[3] Ku E., Gassman J., Appel L.J. (2017). BP control and long-term risk of ESRD and mortality. J Am Soc Nephrol.

[4] Alencar de Pinho N., Levin A., Fukagawa M. (2019). Considerable international variation exists in blood pressure control and antihypertensive prescription patterns in chronic kidney disease. Kidney Int.

[5] Taal M.W., Lucas B., Roderick P. (2023). Associations with age and glomerular filtration rate in a referred population with chronic kidney disease: methods and baseline data from a UK multicentre cohort study (NURTuRE-CKD). Nephrol Dial Transplant.

[6] Peterson J.C., Adler S., Burkart J.M. (1995). Blood pressure control, proteinuria, and the progression of renal disease. The modification of diet in renal disease study. Ann Intern Med.

[7] Appel L.J., Wright J.T., Greene T. (2010). Intensive blood-pressure control in hypertensive chronic kidney disease. N Engl J Med.

[8] Cheung A.K., Chang T.I., Cushman W.C. (2021). KDIGO 2021 clinical practice guideline for the management of blood pressure in chronic kidney disease. Kidney Int.

[9] Cheung A.K., Whelton P.K., Muntner P. (2023). International consensus on standardized clinic blood pressure measurement – a call to action. Am J Med.

