# Supplementary material for: Associations With Baseline Blood Pressure Control in NURTuRE-CKD
Source: Kidney Int Rep. 2024 Feb 15;9(5):1508–12. doi: 10.1016/j.ekir.2024.02.013 (PMC11068972; doi:10.1016/j.ekir.2024.02.013)
Supplement: Supplementary File (PDF) [file mmc1.pdf]

Supplementary File

Supplementary Methods

The NURTuRE-CKD cohort study and biorepository includes 2996 participants from 16 nephrology centres across the UK. Detailed methods have been described previously[5]. Eligibility criteria included age ≥18 years, at least one attendance to a nephrology clinic, eGFR 15-59ml/min/1.73m^2 or eGFR ≥60ml/min/1.73m^2 with urine albumin creatinine ratio (UACR) >30mg/mmol, willing to participate in two study visits and able to give informed consent. Solid organ transplant recipients, those receiving chemotherapy or cancer treatment, expected survival <1 year, AKI or a major cardiovascular event within three months of recruitment were excluded. Recruitment commenced in 2017 and was completed in 2019.

The study was approved by the South Central - Berkshire Research Ethics Committee and is registered at Clinical Trials.gov (NCT04084145). NURTuRE is a collaborative project with multiple academic and commercial partners and is governed by a formal collaboration agreement. Funding is provided by the commercial partners (UCB Biopharma, Evotec International GmbH, Astra Zeneca, AbbVie and Travere therapeutics) with all funds paid to Kidney Research UK and awarded to the investigators as a research grant[S1]

A single baseline visit was conducted by trained members of research staff for each participant. Three seated BP readings differing by <10% were taken using an oscillometric device according to a standard operating procedure and the mean of the three systolic and diastolic values calculated.

Diabetes status was defined by self-reported diabetic history, or use of antidiabetic medication. Atherosclerotic cardiovascular disease was defined as any history of

stroke, myocardial infarction, coronary artery bypass surgery, peripheral vascular disease or amputation either self-reported or from medical notes and recorded on the case record form. Sex was self-reported at baseline by participants. Stored samples from the baseline visit were analysed centrally for serum creatinine and urine albumin to creatinine ratio (UACR) at Geneva University Hospitals Switzerland to give a baseline eGFR and UACR for each participant. Estimated glomerular filtration rate (eGFR) was calculated using the CKD-EPI equation (2009) without the ethnicity variable as recommended by NICE. Anti-hypertensive medications were recorded at baseline by the research teams and categorized by their method of action. Preparations containing two agents were separated into medication classes. Data were kept centrally in a database held in the UK Renal Registry (UKRR).

Blood Pressure Control

Baseline mean systolic and diastolic BP values were compared against three guidelines; two applicable to participants at time of recruitment: the 2014 NICE guideline[S2], and the international KDIGO 2012[S3] guideline. NICE recommended different targets by albuminuria and diabetes status (<140/90 mmHg without diabetes, <130/80 mmHg with diabetes or UACR ≥70 mg/mmol) and KDIGO by albuminuria status only (<140/90 mmHg, unless high risk ACR >30 mg/g then <130/80 mmHg. In 2021 KDIGO issued an updated guideline [S4] suggesting a target of <120 mmHg systolic based on findings from the intensive versus standard blood pressure intervention (SPRINT) trial[S1] and BP control was assessed against the lower target to gauge the proportion of the recruited population that would meet it. BP control was defined as systolic and/or diastolic reading at baseline below the recommended target for each.

Statistical Analysis

Continuous data are presented as mean ± standard deviation (SD) where normally distributed and otherwise as median and interquartile range (IQR). Categorical variables are presented as counts and percentages. Comparison of mean systolic and diastolic BP were performed by independent t-test or 1 way analysis of variance (ANOVA).

To investigate factors associated with BP control, univariable logistic regression analysis was performed with achievement of each BP target as the dependent variable. To identify independent associations, multivariable logistic regression analysis for BP control by guideline was performed adjusting for age (<65 years), sex, ethnicity, diabetes, history of atherosclerotic cardiovascular disease, body mass index (BMI), smoking status, employment, number of antihypertensives, renin angiotensin system inhibition (RAASi), albuminuria status and eGFR. Variables to be included in the models were selected based on previously reported associations with BP control[S5-S8] or significant associations in the univariable analyses. Odds ratios are reported with 95% confidence intervals with a p value of <0.05 considered statistically significant.

All statistical analysis was performed using IBM SPSS v 28.01.1

Supplementary Results

Supplementary Table S1. Blood Pressure by baseline demographics

	Patient number (%)	SBP mmHg	DBP mmHg	*P* value SBP	*P* value DBP
**Whole cohort**	2683	140±20	80±12		
**Age years**					
≥65	1384 (52)	143±19	76±12	< 0.001	< 0.001
<65	1299 (48)	136±19	84±12		
**Sex**					
Male	1590 (59)	140±20	80±13	0.015	0.980
Female	1093 (41)	138±21	80±12		
**Ethnicity**					
White	2322 (87)	140±21	80±12	< 0.001	< 0.001
Black	178 (7)	147±22	87±16		
Asian	84 (3)	135±20	82±12		
Other	97 (4)	135±17	81±11		
**eGFR ml/min/1.73m^2**					
>60	271 (10)	135±19	84±11	< 0.001	< 0.001
45-60	458 (17)	136±19	82±12		
30-44	891 (33)	141±21	81±12		
15-29	976 (36)	141±21	78±13		
<15	87 (3)	142±20	77±13		
**Albuminuria mg/g**					
<30	625 (23)	134±19	77±11	< 0.001	< 0.001
≥30-300	877 (33)	138±20	79±12		
≥300	1181(44)	144±21	82±13		
**Diabetes**					
Yes	812 (30)	144±21	76±12	< 0.001	< 0.001
No	1871 (70)	138±20	82±12		
**BMI kg/m^2**					
>30	1071 (40)	141±20	80±13	< 0.001	0.74
25-30	935 (35)	140±20	80±12		
<25	606 (23)	136±21	79±12		
**RAASi**					
Yes	1830 (68)	140±20	81±12	0.491	< 0.001
No	853 (32)	139±21	79±12		
**Smoking status**					
Current	230 (9)	141±19	83±12	0.609	< 0.001
Ex-smoker	1083 (40)	140±21	79±12		
Never smoked	1335 (50)	139±20	81±12		
**History of atherosclerotic CVD**					
Yes	445 (17)	143±23	77±13	0.003	< 0.001
No	2197 (82)	139±20	81±12		
**Primary Renal Disease**					

CKD of uncertain aetiology	816 (30)	141±21	79±12	< 0.001	< 0.001
Glomerular disease	650 (24)	137±18	82±11		
Diabetic kidney disease	310 (12)	147±20	76±12		
Inherited nephropathies	291 (11)	135±17	83±11		
Hypertension/renal vascular disease	242 (9)	144±25	79±15		
Tubulointerstitial disease	155 (6)	137±20	82±15		
Other systemic diseases affecting the kidney	56 (2)	131±27	75±14		
Congenital	45 (2)	137±19	83±12		
Urological	116 (4)	132±18	81±10		
**Educational level**					
No qualifications	685 (25)	143±22	77±12	< 0.001	< 0.001
GCSE	654 (24)	140±21	80±12		
A Levels	204 (8)	139±20	81±13		
NVQ	358 (13)	138±21	82±12		
First degree	442 (16)	137±18	82±11		
Higher degree	282 (11)	136±18	81±11		
Other	16 (0.6)	142 (127-151)	81 (66-89)		
**Health Literacy**					
SILS >2	138 (5)	140±19	80±14	0.936	0.805
SILS ≤2	2477 (92)	140±21	80±12		
**Employment Status**					
Working	926 (35)	135±18	84±11	< 0.001	< 0.001
Retired	1403 (52)	143±21	77±12		
Unemployed	61 (2)	138 (121-151)	82 (77-90)		
Student	10 (0.3)	123 (117-131)	80 (78-83)		
Other	251 (9)	140±22	84 ±13		
**IMD Quintile**					
1 (Most deprived)	575	141±20	80±11	0.184	0.329
2	565	140±20	80±12		
3	507	138±20	80±12		
4	492	139±20	80±12		
5 (Least deprived)	538	139±21	81±13		

BMI- body mass index, CVD – cardiovascular disease, CKD – chronic kidney disease, eGFR – estimated glomerular filtration rate, GCSE – general certificate of secondary education, IMD- index of multiple deprivation, NVQ- national vocational qualification, RAASi - renin angiotensin system inhibition, SILS – single-item literacy screener where a score of >2 suggested impaired health literac

Supplementary Figure S1. Blood pressure in the NURTuRE-CKD cohort according to KDIGO CKD risk categories (n=2683, data are median (IQR))

		A1<br>UACR <30 mg/g	A2<br>UACR 30-300 mg/g	A3<br>UACR >300 mg/g
**G1 (GFR >90ml/min/1.73m^2)**	**SBP**<br>**DBP**	127 (119-150)<br>81 (78-89)	130 (119-151)<br>88 (78-96)	129 (118-138)<br>84 (78-92)
**G2 (GFR 60-89ml/min/1.73m^2)**	**SBP**<br>**DBP**	130 (119-146)<br>81 (75-90)	136 (122-145)<br>84 (75-92)	136 (126-151)<br>85 (77-93)
**G3a (GFR 45-59ml/min/1.73m^2)**	**SBP**<br>**DBP**	132 (121-142)<br>80 (72-87)	134 (123-145)<br>81 (73-88)	140 (127-157)<br>84 (78-91)
**G3b (GFR 30-44ml/min/1.73m^2)**	**SBP**<br>**DBP**	133 (123-143)<br>77 (69-85)	138 (125-153)<br>81 (73-88)	142 (130-156)<br>82 (74-90)
**G4 (GFR 15-29 ml/min/1.73m^2)**	**SBP**<br>**DBP**	132 (121-148)<br>72 (64-80)	135 (124-149)<br>76 (68-82)	144 (132-158)<br>80 (72-89)
**G5 (GFR <15 ml/min/1.73m^2)**	**SBP**<br>**DBP**	128 (122-140)<br>64 (56-75)	135 (127-145)<br>73 (64-80)	142 (130-161)<br>78 (71-91)

Supplementary Figure S2. Mean ± standard deviation BP at baseline by KDIGO CKD risk categories in the NURTuRE CKD cohort study (n=2683)

KDIGO Risk	SBP	DBP
Very low	133±18	82±11
Low	134±18	82±11
Medium	135±19	81±11
High	142±11	80 ±13

DBP – diastolic blood pressure, SBP- systolic blood pressure

Supplementary Figure S3. Proportion of participants meeting KDIG0 2021 BP guideline (systolic BP <120mmHg) by KDIGO GFR category n (%) in NURTuRE CKD at baseline

	A1<br>uACR <30 mg/g	A2<br>uACR 30-300 mg/g	A3<br>uACR >300 mg/g
G1<br>(eGFR >90 ml/min/1.73m^2)	1 (20)	3 (30)	10 (29)
G2<br>(eGFR 60-89 ml/min/1.73m^2)	20 (29)	14 (20)	15 (17)
G3a<br>(eGFR 45-59 ml/min/1.73m^2)	38 (22)	25 (17)	16 (10)
G3b<br>(eGFR 30-44 ml/min/1.73m^2)	43 (19)	48 (16)	24 (7)
G4<br>(eGFR 15-29 ml/min/1.73m^2)	35 (24)	64 (19)	47 (10)
G5<br>(eGFR <15 ml/min/1.73m^2)	1 (14)	0	5 (12)

eGFR – estimated glomerular filtration rate, uACR – urinary albumin creatinine ratio

Supplementary Figure S4. Proportion of participants meeting KDIGO 2012 BP target (<140/90 mmHg, unless high risk ACR >30 mg/g then <130/80 mmHg) in NURTuRE CKD at baseline

	A1<br><br>uACR <30 mg/g	A2<br><br>uACR 30-300 mg/g	A3<br><br>uACR >300 mg/g
G1<br><br>(eGFR >90 ml/min/1.73m^2)	3 (60)	2 (20)	9 (26)
G2<br><br>(eGFR 60-89 ml/min/1.73m^2)	39 (56)	19 (27)	21 (33)
G3a<br><br>(eGFR 45-59 ml/min/1.73m^2)	111 (66)	36 (25)	26 (16)
G3b<br><br>(eGFR 30-44 ml/min/1.73m^2)	137 (60)	70 (23)	568(15)
G4<br><br>(eGFR 15-29 ml/min/1.73m^2)	95 (65)	96 (29)	75 (15)
G5<br><br>(eGFR <15 ml/min/1.73m^2)	4 (57)	4 (31)	9 (21)

eGFR – estimated glomerular filtration rate, uACR – urinary albumin creatinine ratio.

Supplementary Figure S5. Proportion of participants meeting NICE BP target (<140/90 mmHg without diabetes, <130/80 mmHg with diabetes or uACR ≥ 70mg/mmol) in NURTuRE CKD at baseline

	Diabetes	A1<br>uACR <30mg/g	A2<br>uACR 30-300 mg/g	A3<br>uACR <70mg/mmol	A3<br>uACR >70mg/mmol
G1 (eGFR >90 ml/min/1.73m^2)	Yes	*No participants*	0/3 (0)	1 (33)	2 (29)
	No	3 (60)	4 (57)	1 (100)	6 (26)
G2 (eGFR 60-89 ml/min/1.73m^2)	Yes	1 (25)	3 (33)	0 (0)	5 (28)
	No	37 (56)	33 (53)	6 (46)	14 (24)
G3a (eGFR 45-59 ml/min/1.73m^2)	Yes	8 (32)	*5 (22)*	0 (0)	5 (14)
	No	95 (66)	72 (60)	26 (72)	9 (11)
G3b (eGFR 30-44 ml/min/1.73m^2)	Yes	23 (36)	20 (22)	9 (20)	13 (13)
	No	99 (61)	108 (50)	30 (41)	20(13)
G4 (eGFR 15-29ml/min/1.73m^2)	Yes	21 (46)	32 (28)	7 (19)	12 (8)
	No	64 (63)	130 (59)	33 (40)	37 (18)
G5 (eGFR <15ml/min/1.73m^2)	Yes	2 (100)	3 (43)	2 (50)	2 (17)
	No	2 (40)	3 (50)	2 (100)	3 (13)

eGFR- estimated glomerular filtration rate, urinary albumin creatinine ratio uACR.

Supplementary Figure S6. Pattern of use of the three most commonly prescribed antihypertensives at baseline in the NURTuRE-CKD cohort

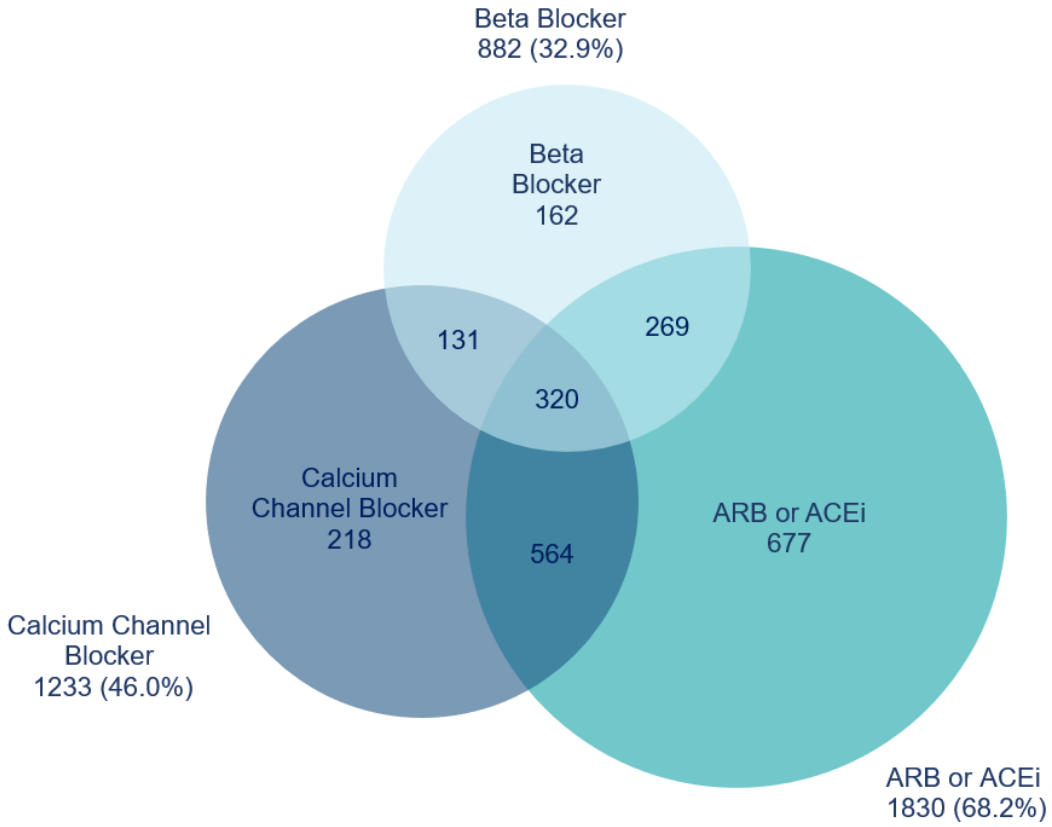

Supplementary Figure S7. Mean systolic and diastolic BP by number of anti-hypertensives in the NURTuRE-CKD cohort

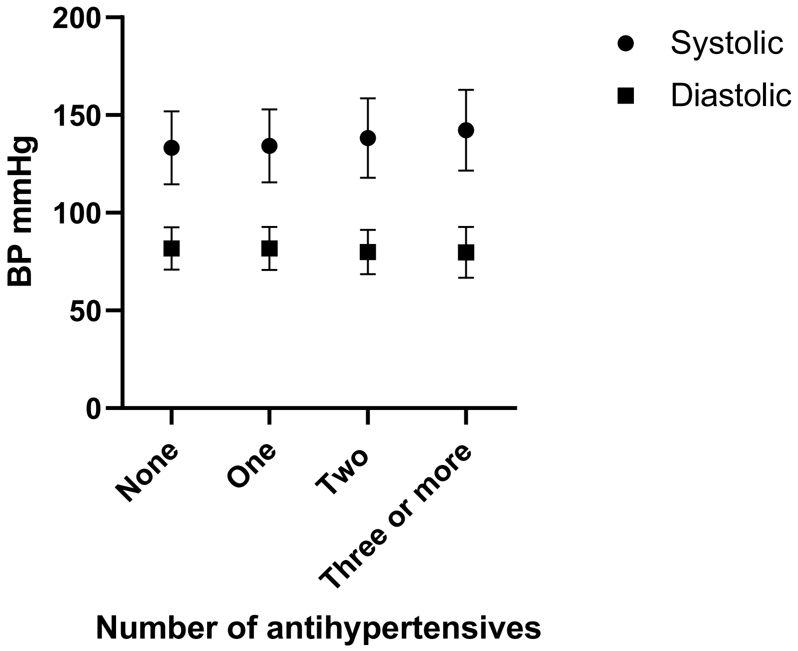

Supplementary Table S2. Univariate associations with BP control by guideline

		**Univariate Odds ratios of achieving KDIGO 2012** OR (95% CI)	*P*	**Univariate Odds ratio of achieving KDIGO 2021** OR (95% CI)	*P*	**Univariate Odds ratio of achieving NICE** OR (95% CI)	*P*
**Age (years)**	≥65	0.913 (0.774,1.077)	0.279	0.578 (0.467,0.716)	< 0.001	0.716 (0.612,0.837)	< 0.001
	<65	Reference		Reference		Reference	
**Sex**	Male	0.731 (0.619,0.683)	< 0.001	0.843 (0.682,1.043)	0.116	0.739 (0.630,0.65)	< 0.001
	Female	Reference		Reference		Reference	
**Ethnicity**	Non-white ethnicity	0.894 (0.699,1.143)	0.372	0.881 (0.653,1.190)	0.409	0.889 (0.705,1.121)	0.320
	White ethnicity	Reference		Reference		Reference	
**Diabetes**	Diabetes	0.735 (0.611,0.884)	0.001	0.657 (0.514,0.840)	< 0.001	0.342 (0.283,0.413)	< 0.001
	No diabetes	Reference		Reference		Reference	
**BMI (m/kg^2)**	>30	0.648 (0.524,0.802)	< 0.001	0.496 (0.380,0.646)	< 0.001	0.536 (0.437,0.658)	< 0.001
	25-30	0.707 (0.569,0.878)	0.002	0.595 (0.456,0.776)	< 0.001	0.688 (0.559,0.846)	< 0.001
	<25	Reference		Reference		Reference	
**Smoking Status**	Ever smoked	0.907 (0.768,1.070)	0.248	0.945 (0.764,1.168)	0.602	0.930 (0.794,1.088)	0.363
	Never smoked	Reference		Reference		Reference	
**History of CVD disease**	Yes	0.982 (0.786,1.227)	0.874	0.996 (0.749,1.324)	0.976	0.795 (0.641,0.987)	0.037
	No	Reference		Reference		Reference	
**Employment**	Working	Reference		Reference		Reference	
	Retired	0.911 (0.762,1.090)	0.308	0.651 (0.518,0.819)	< 0.001	0.704 (0.594,0.835)	< 0.001
	Unemployed	1.033 (0.595,1.794)	0.908	1.334 (0.718,2.479)	0.362	0.745 (0.435,1.277)	0.284
	Student	3.177 (0.890-11.342)	0.075	2.986 (0.834,10.698)	0.093	5.283 (1.116,25.015)	0.036
	Other	0.635 (0.460,0.880)	0.006	0.824 (0.563,1.205)	0.318	0.653 (0.487,0.875)	0.004
**Education status**	No qualifications	Reference		Reference		Reference	
	GCSE	1.115 (0.881,1.411)	0.365	1.150 (0.846,1.562)	0.372	1.265 (1.011,1.583)	0.040
	A Levels	0.988 (0.698.1.400)	0.948	1.244 (0.807,1.917)	0.323	1.231 (0.890,1.704)	0.209
	NVQ	1.030 (0.776,1.366)	0.839	1.430 (1.011,2.023)	0.043	1.172 (0.897,1.532)	0.245
	First degree	1.245 (0.960,1.613)	0.098	1.213 (0.866,1.699)	0.261	1.395 (1.089,1.788)	0.008
	Higher degree	1.422 (1.059.1.910)	0.019	1.224 (0.831,1.802)	0.306	1.688 (1.271,2.241)	< 0.001
	Other	0.850 (0.271,2.667)	0.780	0.430 (0.056,3,292)	0.416	0.923 (0.317,2.689)	0.883)
**IMD Quintile**	1 Most deprived	1.045 (0.807, 1.353)	0.737	0.597 (0.426, 0.837)	0.003	1.132 (0.888,1.444)	0.316
	2	1.108 (0.856, 1.433)	0.436	0.726 (0.524, 1.007)	0.055	1.106 (0.866, 1.413)	0.418
	3	1.048 (0.804, 1.368)	0.727	1.007 (0.734, 1.382)	0.965	1.045 (0.812, 1.344)	0.734
	4	1.137	0.344	0.881	0.446	1.164	0.239

		(0.871, 1.483)		(0.635, 1.221)		(0.904, 1.498)	
	5 (Least deprived)	Reference		Reference		Reference	
**Number of antihypertensives**	None	Reference		Reference		Reference	
	One	0.659 (0.496,0.878)	0.004	1.003 (0.698,1.442)	0.987	0.827 (0.613,1.114)	0.211
	Two	0.576 (0.433,0.767)	< 0.001	0.846 (0.564,1.269)	0.419	0.636 (0.458,0.883)	0.007
	Three or more	0.373 (0.282,0.493)	< 0.001	0.487 (0.352,0.675)	< 0.001	0.447 (0.345,0.579)	< 0.001
**RAASi**	No	Reference		Reference		Reference	
	Yes	0.763 (0.641,0.908)	0.002	0.931 (0.747,1.161)	0.526	0.843 (0.716,0.994)	0.042
**uACR mg/g**	A1	Reference		Reference		Reference	
	A2	0.212 (0.170,0.264)	< 0.001	0.752 (0.581,0.972)	0.029	0.677 (0.551,0.832)	< 0.001
	A3	0.121 (0.097,0.151)	< 0.001	0.388 (0.297,0.508)	< 0.001	0.199 (0.161,0.246)	< 0.001
**eGFR ml/min/1.73m^2**	Per 1ml/min/1.73 m^2	1.007 (1.002,1.011)	0.005	1.009 (1.004-1.015)	0.001	1.007 (1.003-1.011)	0.001

BMI- body mass index, CVD – cardiovascular disease, eGFR – estimated glomerular filtration rate, GCSE – general certificate of secondary education, IMD- index of multiple deprivation, NVQ- national vocational qualification, RAASi - renin angiotensin system inhibition, UACR – urinary albumin creatinine ratio

Supplementary References

S1 Wright JT, Jr., Williamson JD, et al. A Randomized Trial of Intensive versus Standard Blood-Pressure Control. *N Engl J Med*. Nov 26 2015;373(22):2103-16. doi:10.1056/NEJMoa1511939

S2 National Institute of Health and Care Excellence (NICE). Chronic kidney disease: assessment and management. ed2014.

S3 Kidney Disease: Improving Global Outcomes Chronic Kidney Disease Guideline Development Work Group M. KDIGO 2012 Clinical Practice Guideline for the Evaluation and Management of Chronic Kidney Disease. Guideline. *Kidney International*. 2013 2013;3(1)

S4 Cheung AK, Chang TI, Cushman WC, et al. KDIGO 2021 Clinical Practice Guideline for the Management of Blood Pressure in Chronic Kidney Disease. Kidney International. 2021/03/01/ 2021;99(3, Supplement):S1-S87. doi:https://doi.org/10.1016/j.kint.2020.11.003

S5 Schneider MP, Hilgers KF, Schmid M, et al. Blood pressure control in chronic kidney disease: A cross-sectional analysis from the German Chronic Kidney Disease (GCKD) study. *PLoS One*. 2018;13(8):e0202604. doi:10.1371/journal.pone.0202604

S6 Muntner P, Anderson A, Charleston J, et al. Hypertension awareness, treatment, and control in adults with CKD: results from the Chronic Renal Insufficiency Cohort (CRIC) Study. *Am J Kidney Dis*. Mar 2010;55(3):441-51. doi:10.1053/j.ajkd.2009.09.014

S7 Alencar de Pinho N, Levin A, Fukagawa M, et al. Considerable international variation exists in blood pressure control and antihypertensive prescription patterns in chronic kidney disease. *Kidney Int*. Oct 2019;96(4):983-994. doi:10.1016/j.kint.2019.04.032

S8 Fraser SD, Roderick PJ, McIntyre NJ, et al. Suboptimal blood pressure control in chronic kidney disease stage 3: baseline data from a cohort study in primary care. *BMC Fam Pract*. Jun 24 2013;14:88. doi:10.1186/1471-2296-14-88

S9 Thomas G, Xie D, Chen HY, et al. Prevalence and Prognostic Significance of Apparent Treatment Resistant Hypertension in Chronic Kidney Disease: Report From the Chronic Renal Insufficiency Cohort Study. *Hypertension*. Feb 2016;67(2):387-96. doi:10.1161/HYPERTENSIONAHA.115.06487

S10 Fraser SD, Taal MW. Multimorbidity in people with chronic kidney disease: implications for outcomes and treatment. Curr Opin Nephrol Hypertens. Nov 2016;25(6):465-472. doi:10.1097/MNH.0000000000000270

S11 Schmidt IM, Hubner S, Nadal J, et al. Patterns of medication use and the burden of polypharmacy in patients with chronic kidney disease: the German Chronic Kidney Disease study. Clin Kidney J. Oct 2019;12(5):663-672. doi:10.1093/ckj/sfz046

S12 Schmitt KE, Edie CF, Laflam P, Simbartl LA, Thakar CV. Adherence to antihypertensive agents and blood pressure control in chronic kidney disease. Am J Nephrol. 2010;32(6):541-8. doi:10.1159/000321688

STROBE Statement—checklist of items that should be included in reports of observational studies

	Item No	Recommendation	Page No
**Title and abstract**	1	(*a*) Indicate the study's design with a commonly used term in the title or the abstract	1
		(*b*) Provide in the abstract an informative and balanced summary of what was done and what was found	N/A
**Introduction**			
Background/rationale	2	Explain the scientific background and rationale for the investigation being reported	1
Objectives	3	State specific objectives, including any prespecified hypotheses	1
**Methods**			
Study design	4	Present key elements of study design early in the paper	Supplementary 1
Setting	5	Describe the setting, locations, and relevant dates, including periods of recruitment, exposure, follow-up, and data collection	Supplementary 1,2
Participants	6	(*a*) *Cohort study*—Give the eligibility criteria, and the sources and methods of selection of participants. Describe methods of follow-up<br>*Case-control study*—Give the eligibility criteria, and the sources and methods of case ascertainment and control selection. Give the rationale for the choice of cases and controls<br>*Cross-sectional study*—Give the eligibility criteria, and the sources and methods of selection of participants	Supplementary 1,2
		(*b*) *Cohort study*—For matched studies, give matching criteria and number of exposed and unexposed<br>*Case-control study*—For matched studies, give matching criteria and the number of controls per case	
Variables	7	Clearly define all outcomes, exposures, predictors, potential confounders, and effect modifiers. Give diagnostic criteria, if applicable	
Data sources/ measurement	8*	For each variable of interest, give sources of data and details of methods of assessment (measurement). Describe comparability of assessment methods if there is more than one group	Supplementary 3
Bias	9	Describe any efforts to address potential sources of bias	4
Study size	10	Explain how the study size was arrived at	
Quantitative variables	11	Explain how quantitative variables were handled in the analyses. If applicable, describe which groupings were chosen and why	

Statistical methods	12	(*a*) Describe all statistical methods, including those used to control for confounding	Supplementary 3
		(*b*) Describe any methods used to examine subgroups and interactions	
		(*c*) Explain how missing data were addressed	Supplementary 3
		(*d*) *Cohort study*—If applicable, explain how loss to follow-up was addressed<br>*Case-control study*—If applicable, explain how matching of cases and controls was addressed<br>*Cross-sectional study*—If applicable, describe analytical methods taking account of sampling strategy	
		© Describe any sensitivity analyses	

Results

Participants	13*	(a) Report numbers of individuals at each stage of study—eg numbers potentially eligible, examined for eligibility, confirmed eligible, included in the study, completing follow-up, and analysed	
		(b) Give reasons for non-participation at each stage	
		© Consider use of a flow diagram	
Descriptive data	14*	(a) Give characteristics of study participants (eg demographic, clinical, social) and information on exposures and potential confounders	Supplementary table 1
		(b) Indicate number of participants with missing data for each variable of interest	Supplementary table 1
		(c) *Cohort study*—Summarise follow-up time (eg, average and total amount)	
Outcome data	15*	*Cohort study*—Report numbers of outcome events or summary measures over time	
		Case-control study—Report numbers in each exposure category, or summary measures of exposure	
		Cross-sectional study—Report numbers of outcome events or summary measures	
Main results	16	(*a*) Give unadjusted estimates and, if applicable, confounder-adjusted estimates and their precision (eg, 95% confidence interval). Make clear which confounders were adjusted for and why they were included	Supplementary table 2
		(*b*) Report category boundaries when continuous variables were categorized	Supplementary methods and tables
		(*c*) If relevant, consider translating estimates of relative risk into absolute risk for a meaningful time period	
Other analyses	17	Report other analyses done—eg analyses of subgroups and interactions, and sensitivity analyses	

Discussion

Key results	18	Summarise key results with reference to study objectives	4
Limitations	19	Discuss limitations of the study, taking into account sources of potential bias or imprecision. Discuss both direction and magnitude of any potential bias	4

Interpretation	20	Give a cautious overall interpretation of results considering objectives, limitations, multiplicity of analyses, results from similar studies, and other relevant evidence	4
Generalisability	21	Discuss the generalisability (external validity) of the study results	4
**Other information**			
Funding	22	Give the source of funding and the role of the funders for the present study and, if applicable, for the original study on which the present article is based	Supplementary methods
